# Antifungal Treatment in Stem Cell Transplantation Centers in Turkey

**DOI:** 10.4274/tjh.2014.0012

**Published:** 2016-02-17

**Authors:** Hamdi Akan, Erden Atilla

**Affiliations:** 1 Ankara University Faculty of Medicine, Department of Hematology, Ankara, Turkey

**Keywords:** Antifungal treatment, diagnosis, stem cell transplantation

## Abstract

Despite the development of various guidelines, the approach to antifungal treatment in stem cell transplantation centers differs according to country or even between centers. This led to the development of another survey that aims to understand the antifungal treatment policies of Turkish stem cell transplantation centers. Although there has been an increasing trend towards the use of diagnostic-based treatments in Turkey in the last few years, empirical treatment is still the main approach. The practices of the stem cell transplantation centers reflect the general trends and controversies in this area, while there is a considerable use of antifungal combination therapy.

## Abstract (tr)

Çeşitli kılavuzlara rağmen, antifungal hastalıkların tedavisine yaklaşım kök hücre nakil merkezlerinde ülkeden ülkeye, hatta aynı ülke içerisinde farklı merkezlerde farklılık göstermektedir. Bu farklılıkları belirlemek amacı ile ilk defa 2010 yılında Türkiye’deki kök hücre nakli merkezlerinde profilaksi yaklaşımlarını anlamak üzere bir anket düzenlemiştik. Bu anket, Türkiye’deki merkezlerde tedavi yaklaşımlarını anlamamıza yol açacak yeni bir çalışma yapmamızı sağladı. Genel olarak tanı-güdümlü yaklaşım giderek artma eğilimi gösterse de, ampirik yaklaşım hala ilk seçenektir. Kök hücre nakli merkezlerindeki yaklaşımlar genel eğilimlere ve tartışmalar uygun gözükse de, kombine antifungal kullanımının yaygın olduğu görülmektedir.

## INTRODUCTION

Despite the development of various guidelines [1,2,3], approaches to antifungal treatment in stem cell transplantation (SCT) centers differ according to country and even between centers. This inspired the development of another survey aimed at understanding the antifungal treatment policies of Turkish SCT centers.

## MATERIALS AND METHODS

**Figure 1:**
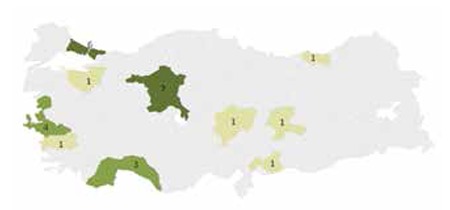
Distribution of stem cell transplantation centers responding to the query.

Out of 28 EBMT-registered SCT centers, 26 responded to the survey (Figure 1). The questionnaire consisted of separate sections defined to understand the basic treatment approach in each center as empirical or diagnostic-driven, the use of diagnostic tools to start or end a treatment, strategies in empirical or diagnostic-driven treatment, and the use of antifungal combinations.

## RESULTS

### Center Characteristics

While 19 (73.1%) of these centers are adult SCT centers, 7 (26.9%) are pediatric SCT centers. While all centers (26) are performing allogeneic transplants, 24 centers are also performing autologous transplants. Among the 26 allogeneic centers, 24 are performing non-myeloablative, 7 non-related, and 6 cord blood cell transplants.

### Treatment Approach

**Figure 2:**
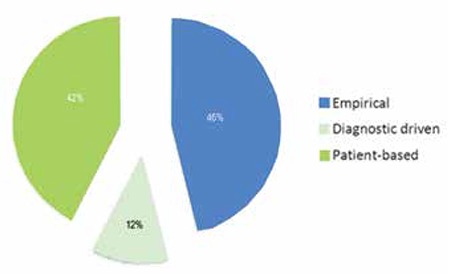
Approach to the treatment of invasive fungal disease in stem cell transplantation centers.

Four centers (16%) reported that they were only using empirical antifungal treatment, while 56% of the centers reported that they initially employ empirical treatment but that further treatment decisions are based on diagnostic tools such as high resolution computed tomography (HRCT) of the lungs and galactomannan (GM) (Figure 2). Twenty percent of the centers reported that they always use a diagnostic-driven approach and 8% of the centers stated that they use an empirical approach in selected cases.

### Salvage Therapy

In non-responding patients, 70% of the centers stop the initial antifungal treatment and switch to another class of antifungal. Twenty-five percent of the centers reported that they add another antifungal to the initial treatment.

### Drug Selection

**Figure 3:**
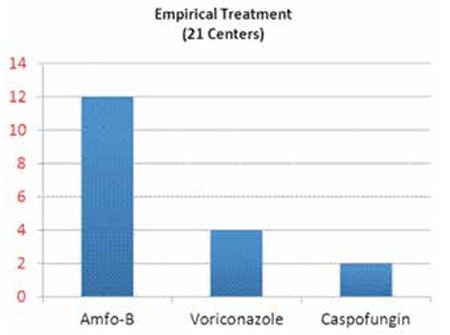
The initial antifungal used for empirical treatment in stem cell transplantation centers.

In empirical approaches, the first drug is amphotericin-B (conventional in 6/21 centers, liposomal in 6/21 centers) in the allogeneic setting (Figure 3). This is followed by voriconazole (4/21) and caspofungin (2/21). This trend is similar in the autologous setting, but voriconazole is less commonly used in autologous transplants. Voriconazole is the main choice in proven cases in allogeneic (23/25) and in autologous (21/23) transplants.

In centers treating their patients based on HRCT and GM (diagnostic-driven treatment), the main drug of choice is voriconazole (15/20), followed by amphotericin-B (5/20), in the allogeneic setting. This trend is similar in other transplant settings.

**Figure 4:**
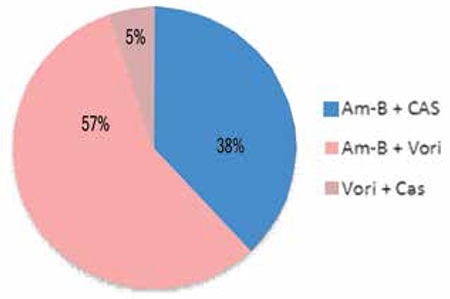
The antifungal combinations used in centers giving salvage treatment (25% of all centers) (Am-B: amphotericin-B, CAS: caspofungin, Vori: voriconazole).

When using antifungal combination therapy, 57% of the centers add voriconazole to initial amphotericin-B treatment, while 38% add caspofungin to initial amphotericin-B and 5% use voriconazole with caspofungin (Figure 4).

### End of Treatment

It was found that 33.3% of the centers continue the antifungal treatment until the end of neutropenia in empirical treatment. Other centers reported that they use both resolution of neutropenia and other evidence such as the clinical condition, diagnostic tools, presence of graft-versus-host disease (GVHD), and pre-transplant fungal status to decide to stop the treatment.

In diagnostic-driven approaches, treatment mainly stops at 90 days (23.8%), after radiological improvement (19%), or after resolution of neutropenia (14.3%).

Most of the centers continue oral antifungals, especially in patients with partial radiological resolution and GVHD.

### Patient Selection

Diagnostic-driven treatment is mainly used in allogeneic settings (19/26 in allogeneic transplants, 18/24 in nonmyeloablative transplants), with a rate of 62.5% (15/24) in autologous settings.

### Candida Treatment

Echinocandin is the first drug of choice in established Candida infections at 17/25 centers in allogeneic and 17/23 centers in autologous transplants, followed by amphotericin-B (5/25) in allogeneic and fluconazole in autologous settings.

### Further Treatment

In patients not responding to initial antifungal treatment, 70% of the centers stop the initial antifungal and start a new one, and 25% of the centers choose to use a combination antifungal treatment.

### Diagnostic Tools

HRCT is routine in 23/26 centers, GM is routine in 4/26 centers, and beta-glucan and molecular diagnosis are routine in 4/26 centers.

### General Approach

When asked about their view on empirical or diagnostic-driven approaches in patients with prolonged fever and neutropenia, 46.2% responded in favor of empirical treatment and 11.5% in favor of a diagnostic-driven approach, while 42.3% responded that the choice should be made per patient and most of them choose to use empirical treatment in high-risk patients (Figure 2).

## DISCUSSION AND CONCLUSION

Although there has been an increasing trend towards the use of diagnostic-based treatments in Turkey in the last few years, empirical treatment is still the main approach. HRCT is the major determinant of diagnostic-driven treatment, and while amphotericin-B is the main drug in empirical treatment, voriconazole is the main choice in diagnostic-driven treatment and proven cases. Despite the guidelines, a large number of centers are using antifungal drug combinations. Keeping in mind that there is still controversy about the selection of empirical therapy versus preemptive (diagnostic-driven) therapy, the differences between the centers in this aspect is understandable. The frequent use of antifungal combinations is interesting, but especially in centers with inadequate diagnostic tools, this approach is to be expected, especially when physicians are faced with a fungal infection that may end up having dreadful consequences.
